# Assessing the health consequences of northern Ethiopian armed conflict, 2022

**DOI:** 10.1057/s41271-023-00464-z

**Published:** 2024-02-03

**Authors:** Mulugeta Wodaje Arage, Henok Kumsa, Mulu Shiferaw Asfaw, Abebe Tarekegn Kassaw, Ephrem Mebratu, Abayneh Tunta, Woldeteklehymanot Kassahun, Amanuel Adissu, Molla Yigzaw, Tilahun Hailu, Lebeza Alemu Tenaw

**Affiliations:** 1 School of Midwifery, College of Health Sciences, Woldia University, North Wollo, Amhara Region Ethiopia; 2 School of Medicine, College of Health Sciences, Woldia University, North Wollo, Amhara Region Ethiopia; 3 Department of Pharmacy, College of Health Sciences, Woldia University, North Wollo, Amhara Region Ethiopia; 4 Department of Pharmacy, College of Medicine and Health Sciences, Debre Markos University, Debre Markos, Amhara Region Ethiopia; 5 Department of Medical Laboratory, College of Health Sciences, Woldia University, North Wollo, Amhara Region Ethiopia; 6 Department of Public Health, College of Health Sciences, Injibara University, Injibara, Amhara Region Ethiopia; 7 Department of Public Health, College of Health Sciences, Debre Markos University, Debre Markos, Amhara Region Ethiopia; 8 School of Public Health, College of Health Sciences, Woldia University, North Wollo, Amhara Region Ethiopia

**Keywords:** Armed conflict, Health impact, Coping mechanisms, Mixed study, Northern Ethiopia

## Abstract

Armed conflict is a complicated topic with multidimensional impact on population health. This study aimed to assess of the health consequences of the northern Ethiopian conflict, 2022. We used a mixed method study design with a retrospective cross-sectional study supplemented by a qualitative study conducted from May to June 2022. We interviewed 1806 individuals from 423 households and conducted 100 in-depth interviews and focused group discussion. We identified 224 people who self-reported cases of illness (124/1000 people) with only 48 (21%) people who fell ill visited a health institution. We also detected 27 cases of deaths (15/1000 people) during the conflict. The collapse of the health system, evacuation of health personnel, and shortage of medical supplies, and instability with a lack of transportation were consequences of the conflict. The northern Ethiopian conflict has greatly affected the community’s health through the breakdown of the health system and health-supporting structures.

**Supplementary Information:**

The online version contains supplementary material available at 10.1057/s41271-023-00464-z.

## Key Messages

- The conflict in Northern Ethiopia has had a substantial impact on the communities health, both directly through conflict-related causalities and indirectly through the disintegration of the health system and health-supporting structures, which has severely hampered the supply and utilization of health services.
- The main coping strategies of patients and health providers for the breakdown of the health system included the use of traditional medications and home remedies, obtaining medications from non-conflict-affected areas, and providing home-to-home service by health providers and using available supplies.
- The study recommended immediate rehabilitating interventions for affected individuals and damaged health infrastructure.

**Supplementary Information:**

The online version contains supplementary material available at 10.1057/s41271-023-00464-z.

## Introduction

The northern Ethiopian conflict, which spanned the northern area, started on November 3, 2020 G.C. in the Tigray Regional state, when Tigray People Liberation Front (TPLF) forces attacked the federal military bases in the region as claimed by the government, while the latter claimed the opposite[1, 2]. Since then, the war has continued in Tigray regional state for two years, and by late June 2021, the conflict had expanded to the neighboring Afar and Amara regional states of northern Ethiopia, where several sources reported many atrocities to civilians and infrastructure destructions [3–6]. Armed conflict leads to a broad range of health consequences, direct or indirect. Direct consequences include morbidity and mortality caused by bullets and bombs. Evidence indicates that conflict-related causalities, both fatal and non-fatal, have caused high morbidity and mortality in both military personnel and non-combatant civilians [7]. An estimated 133,750 individuals are killed annually by conflicts-related violent [8, 9]. According to data from the Violation Documentation Center, a dataset that records violent deaths in Syria from 2011 to 2012, 70% of all conflict-related deaths were civilians [10, 11]. Indirectly, conflict also causes breakdown of a breakdown of the health system, shortage of medical supplies, migration, and even death of health care workers (HCWs), as well as ruptures of food and clean water supplies, which deteriorates the health of the population. Conflict-associated insecurity and a lack of free movement reduce the provision and utilization of health services [12–15]. Conflict also causes a huge spike of humanitarian crisis with many civilians become displaced and many livings in refuges. Exposing the populations at a greater risk of disease and illness, mainly communicable and infectious disease [16–18].

Despite these major impacts of conflict on health and the health system, measuring and estimating the magnitude of the impact of armed conflict on population health is challenging. The fundamental challenge is in conflict settings, health information systems that record events and causes of death frequently cease to function.[19–21]. Available information may also be politicized and intentionally misrepresented [19, 20]. To address these issues, the World Health Organization (WHO) passed a resolution in 2012 that calls for leadership in documenting evidence of attacks against health workers, facilities, and patients in armed conflict settings [19, 22]. In the northern Ethiopian conflict, there have been numerous reports of destruction and atrocities against health systems and civilians [3–5]. However, the majority of these reports have been based on statements made by the fighting parties, as well as press reports from eyewitness accounts, journalists, and humanitarian agencies. Therefore, this shows the necessity of a study that clearly provides information based on scientific data. The current study, which aims at assessing the impact of the northern Ethiopian armed conflict on population health, will provide a clear image of the health-related consequences of the conflict, highlighting the burden of these problems from the perspective of individuals and communities using both quantitative and qualitative components. The findings provide insight for humanitarian interventions and input for the government and other stakeholders in designing empirical and evidence-based health interventions for conflict-affected areas. It will also serve as baseline evidence for further investigation in the area.

## Data and methods

### Study design

We conducted a retrospective cross-sectional study supplemented by qualitative study on conflict-affected areas of North Wollo zone of Amhara regional state to assess the health consequences of the northern Ethiopian conflict during the six-month period of TPLF occupation of the area. The area was under the control of TPLF fighters for about six months from late June to early January. Located about 521 km from Addis Aba, the capital city of Ethiopia, the North Wollo Zone is one of 10 zones in the Amhara region. The conflict started in the Tigray Region and remained confined there for 2 years until it spread north to the zone that borders it. This north Wollo zone encompasses 14 woredas (the third level of administrative division after zones and regional states, which is equivalent to a district) and 313 kebeles (the smallest administrative division, which is comparable to a municipality or village at the neighborhood level) with a total population of 1,500,303 and 355,974 households. The study conducted from May to June 2022 G.C.

We recruited 12 trained health professionals from the respective woredas to conduct the interviews using a structured checklist. The interviewers used an in-depth interview guide and a tape recorder to facilitate these conversations. To ensure the validity and clarity of the checklist, we conducted a pre-test prior to data collection. We also provided training on the study objectives, data collection, and confidentiality. As the location was in a post-conflict area, to avoid unprecedented accidents and for the safety of the interviewers, before starting data collection, the study team provided information about the data collection to the public at communities gatherings.

### Quantitative component

We determined the total sample size for the quantitative study using a single population proportion formula with the assumptions of prevalence: 50%, margin of error: 5%, and a 95% CI and 10% non-response rate of 423. We included in the study households in the area that had individuals living in them during the time of TPLF occupation. We excluded those located in the same areas with no individual residing during the study period and individuals who fled the area during the time of the invasion and returned after its liberation. Using a multi-stage sampling technique, we selected a total of 423 households. First, we randomly (by lottery) selected six of 14 woredas: Guba Lafto, Habru, Kobo, Lasta, Wadla and Woldia woredas. Then, from each of the selected woredas, we randomly selected one kebele. Finally, we selected households from the chosen kebeles using a systematic sampling technique.

We used a structured checklist that included information on the socio-demographic characteristics, the number of self-reported cases of illness or adverse health condition and known cases of death among family members, the causes of illness and death, healthcare seeking options used during episodes of sickness, the type of health service visit, and the reason for not visiting. We also ask about the use of traditional medications and home remedies, obtaining medications from non-conflict-affected areas, and providing home-to-home service by health providers and available supplies.

We collected general information from 1806 individuals representing 423 households, with a response rate of 100%. The mean age of the participants was 23.8 ± 18.3 years. Nearly one-third of the participants were under the age of 15. Female participants constitute near half (51.4%) of the respondents (Table 1).

**Table 1:** Socio-demographic characteristics of 1806 quantitative study participants in northern Ethiopia, 2022

Variables	Frequency (%)
Age	
< 15	596 (33.0)
15–24	355 (19.7)
25–34	301 (16.7)
35–44	288 (15.9)
≥ 45	266 (14.7)
Sec	
Female	928 (51.4)
Male	878 (48.6)
Residence	
Urban	671 (37.2)
Rural	1135 (62.9)
Religion	
Orthodox	1182 (68.5)
Muslim	600 (31.4)
Others	24 (0.1)
Marital status	
Married	678 (37.5)
Single	1008 (55.8)
Divorced	73 (4.0)
Windowed	47 (2.6)
Educational status	
No formal education	887 (49.1)
Primary education	594 (32.9)
High school and preparatory school	221 (12.2)
College and above	104 (5.8)

We entered the recorded data into Epidata version 4.6 and used SPSS version 27.0 software for the analyses. We estimated descriptive statistics (means and frequencies for continuous and categorical variables, respectively) and estimated 95% confidence interval was reported for the proportion of outcomes as shown in Tables 1, 2, 3, 4, 5.

**Table 2:** Composition and characteristics of 100 qualitative study participants in northern Ethiopia, 2022

Respondent type	Focus group discussion	In-depth interview	Total
Individuals who have been sick or have sick family member during the conflict	15	12	27
Patients with chronic diseases	5	4	9
Elders, communities and religious leaders	10	4	14
Member of different communities associations	10	4	14
Health care professionals	8	9	17
Health institution administrators	2	3	5
Pregnant woman	10	4	14

**Table 3:** Self-reported responses on health conditions among 1806 study participants in northern Ethiopia, 2022

Variable	Frequency	Percentage (95% CI)
Have you ever been sick during the conflict?		
Yes	224	12.4 (11.0–13.9)
No	1582	87.6 (86.1–89.0)
Cause of illness		
Medical illness related	215	96.0 (93.7–98.3)
Conflict causality	9	4.0 (1.7–6.3)
Bullet shot	3	1.3 (0.7–2.1)
Artillery shelling	6	2.7 (1.0–4.2)
Have you lost a family member during the 6-month conflict?		
Yes	27	1.5 (0.9–2.1)
No	1779	98.5 (97.9–99.1)
Cause of death		
Medical illness related	23	85.2 (74.8–95.4)
Conflict causality	4	14.8 (4.6–25.2)
Bullet shoot	1	3.7 (1.1–6.4)
Artillery shelling	3	11.1 (3.5–18.8)

**Table 4:** The distribution of self-reported illness and known cases of death among family members during the northern Ethiopian conflict among the 1806 study participants, 2022

Variables	Self-reported cases of illness during the conflict		known cases of death among family during the conflict	
	Yes	No	Yes	No
Age				
< 15	108 (48.2%)	488 (30.8%)	11(40.7%)	585 (37.0%)
15–24	35 (15.6%)	320 (20.2%)	2 (7.4%)	353 (22.3%)
25–34	21 (9.4%)	17.7 (%)	4 (14.8%)	297 (18.8%)
35–44	27 (12.1%)	16.5 (%)	4 (14.8%)	284 (15.3%)
≥ 45	33 (15.2%)	233 (14.8%)	6 (22.2%)	260 (16.4%)
Sex				
Female	116 (51.8%)	812 (52.4%)	15 (55.6)	913 (57.8%)
Male	108 (48.2%)	770 (47.6%)	12 (44.4)	866 (54.7%)
Residence				
Urban	98 (43.8%)	573 (36.2%)	9 (33.3%)	662 (41.8%)
Rural	126 (56.2%)	1009 (63.8%)	18 (66.7%)	1117 (70.6%)
Religion				
Orthodox	139 (62.0%)	1043 (65.9%)	20 (74.0%)	1162 (65.3%)
Muslim	81 (36.2%)	519 (32.8%)	7 (26.0%)	593 (33.3%)
Others	4 (1.8%)	20 (1.3%)	0 (3.7%)	24 (1.3%)
Marital status				
Married	46 (20.5%)	632 (39.9%)	3 (11.1%)	675 (42.7%)
Single	141 (62.9%)	867 (54.8%)	16 (59.3%)	992 (62.7%)
Divorced	15 (6.7%)	58 (3.7%)	3 (11.1%)	70 (4.4%)
Windowed	22 (9.2%)	25 (1.6%)	5 (18.5%)	42 (26.5%)
Educational status				
No formal education	95 (42.4%)	492 (31.1%)	14 (51.9%)	873 (49.1%)
Primary education	98 (43.8%)	696 (44.0%)	8 (29.6%)	586 (32.9%)
High school and preparatory	23 (10.3%)	298 (18.9%)	4 (14.8%)	217 (12.2%)
College and above	8 (3.6%)	96 (6.1%)	1 (3.7%)	103 (5.8%)

**Table 5:** Health service utilization among 224 responders in northern Ethiopia, 2022

Variables	Frequency	Percentage (%)
Health facilities visit during sickness?		
Yes	48	21.4 (17.5–37.9)
No	176	78.6 (66.1–82.5)
Reason for not visiting health facilities		
My condition was not serious	35	19.9 (13.1–25.8)
No functional health institution in the area	74	42.0 (34.9–50.8)
Insecurity	31	17.6 (10.1–24.0)
Belief that poorly staffed and supplied hospitals were unlikely to provide adequate care	24	13.6 (9.2–18.9)
Lack of capacity (financial and supportive person) to visit health facilities	12	6.7 (4.3–9.7)
Satisfaction after visiting health facilities		
Yes	27	56.3 (44.1–63.0)
No	21	43.7 (37.0–55.9)
Reason for lack of satisfaction		
Not receiving adequate care	16	76.2 (65.5–87.9)
Due to absence of a health professional	5	23.8 (18.2–29.4)
Due to lack of medication	11	52.4 (47.3–59.5)
No improvement in disease	3	14.3 (8.5–18.6)
Other (long waiting hour)	2	9.5 (3.6–15.9)

### Qualitative component

For the qualitative study, we conducted in-depth interviews and focus group discussions with purposefully selected key informants. Using a multi-stage sampling technique, we selected a total of 100 respondents and conducted 40 in-depth interviews and 6 focus groups with 10 individuals in each group.

The summary characteristics of 100 respondents, including 40 people participated in-depth interviews and 60 people participated in 6 focus groups (10 individuals in each) is shown in Table 2. These groups included at least one representative from each key informant for the 5 groups, except for health professionals, who all together made up the remaining group (Table 2).

Two investigators separately transcribed the recorded data and translated it into English. After comparing each translation and checking the consistency, we entered the data, coded, and analyzed them using open code version 4.03. We employed a thematic analysis approach to interpret the data. We assembled selected quotes that addressed the study objectives and provided the narratives about the topics of the interview (Supplemental Material Table S1).

### Ethical considerations

The research team has conducted the study after obtaining Ethical approval from the Institutional Review Board (IRB) of Woldia University with protocol number WDU/IRB001. In addition, for the quantitative study, informed consent obtained from the head of household or spouse after explaining the purpose of the study. For information about individuals below the age of 18, we obtained consent from their parents or guardian. Moreover, written informed consent obtained from each qualitative study respondent. Data confidentiality assured by omitting any personal identifiers.

## Results

The study indicated that the conflict caused the health condition of the communities to deteriorate. Out of 1806 participants surveyed, 224 individuals (12.4%) reported being sick at least once. Among the reported sickness, 215 (96%) responders attributed it to the common medical conditions including pediatric diseases, respiratory diseases, malaria, typhoid fever, and chronic diseases, although not confirmed. The study also reported 27 cases of death among immediate family members during the active war period, equivalent to in a death rate of 15 per 1000 people (Table 3).

The male to female ratio of morbidity and mortality were 1.07 (116 males vs. 108 females) and 1.25 (15 males vs. 12 females) respectively. Among the total reported deaths, 40.7% occurred in those under the age of 15 years (Table 4).

The qualitative responses provided additional support to the findings: respondents reported a high toll of morbidities and mortalities during the conflict. In this way we also identified the mechanisms through which the conflict affected the communities health, and we categorized them thematically as shown below.

- Direct conflict-related casualties: Respondents identified conflict-related fatal and non-fatal injuries caused by gunshots and artillery shootings as major factors that contributed to the health crisis and deteriorated the communities health condition. The testimony of one health extension worker in one of the conflict front areas is best described by citation #1 in Table S1.
- Increased infectious and communicable disease: In addition to the direct conflict-related morbidity and mortality, the conflict has inflicted a high rate of communicable and infectious disease. This was caused by the disruption of fundamental disease control measures such as immunization, sanitation, and safe drinking water. It was also associated with limited access to food and nutrition (#2 in Table S1).
- Disruption of social support structures: The conflict indirectly affected community’s health by destroying social support structures. It reduces access to clean water, sanitation, and electricity. This further increases the risk of contracting communicable diseases (#3 in Table S1).
- Food insecurity and malnutrition: During the conflict, there was a widespread famine and food insecurity, which led to hunger and malnutrition (#4 in Table S1).
- Displacement of the population: The conflict-related instability caused civilians to leave their homes in search of safety and to avoid being forced to become militants. Exposing them for a greater risk of disease and illness. The displacement of adults and family members also leaves elders and people in need without a supportive and caring person (#5 in Table S1).
- Sexual violence and rape: The conflict has resulted in a high level of sexual violence and rape with serious health consequences for the survivor and their family (#6 in Table S1). The lack of immediate care and support, together with the social isolation and stigma, exposed the survivors for severe medical and psychological consequences (#7 in Table S1).
- Breakdown of health system: The conflict has caused disruption in the health system, which ranges from the partial or complete shutdown of medical institutions to a shortage of health workers and medical supplies. This exacerbated the poor health conditions in communities (#8 in Table S1).
- Closure of health facilities: The conflict has resulted in the closure of most health facilities in the area, leaving only two hospitals and three health centers to provide services (#9 in Table S1).
- Shortage of medical supplies: The conflict-disrupted health facility supply networks, combined with the widespread looting of medical supplies, led to a pharmaceutical scarcity (#10 & #11 in Table S1).
- Fleeing of healthcare workers: The conflict-related insecurity also caused the fleeing of many healthcare workers, leaving the communities without caretakers(#12 in Table S1).
- Insecurity and lack of transportation: Conflict-associated instability and lack of transportation have substantially affected health provision and utilization. This affected both health workers and patients, all of whom had difficulty getting to health facilities (#13 & #14 in Table S1).
- Overtaking of health facilities by combatants: Combatants forced some health facilities to use their limited medications and supplies to treat wounded fighters (#15 in Table S1).

A large portion (78.6%) of individuals who reported that they felt ill during the conflict (176 out of 224) did not seek medical assistance from any health institution, possibly due to the cumulative effect of the aforementioned factors (Table 4).

Individuals, communities, and healthcare workers implemented various coping measures to lessen the negative health consequences of the conflict-related health challenges, as described below.

- Coping measures taken by patients: To address the lack of access to necessary treatment and medications for their illnesses. On this issue, the most reported measures taken by patients involved the utilization of traditional medicine and home remedies, followed by travel to conflict-free areas (#16 in Table S1). Consistent with the qualitative report, the quantitative survey found that 38.4% of individuals who became ill during the conflict used traditional medicine and home remedies as treatment options (Figure S1).
- Coping measures taken by communities and religious leaders’: To limit the conflict’s health impact, communities and religious leaders, together with the peace committees formed by voluntary communities’ members, have taken different coping mechanisms. These measures include protecting health facilities, encouraging and facilitating the communities to support each other, and bringing medications and supplies from unoccupied areas using available routes (#17 & #18 in Table S1). They also gathered, encouraged, and supported available health workers to provide health service (#19 in Table S1),
- Coping measures taken by healthcare workers: This study showed health workers to have been at the forefront of efforts to cope with the devastating health consequences of the conflict. In response to the closure of health facilities and the shortage of medical supplies, health professionals have showed remarkable resilience and resourcefulness. They have provided home-to-home service and used available materials they could get (#20 & #21 in Table S1). They also provide health education about the importance of boiling the water before use and maintaining personal hygiene to prevent the onset and spread of sporadic diseases from unclean and surface water (#22 in Table S1).
- In some areas, the combatants were also providing service using their medical staff. However, due to a lack of trust in the communities there were only a limited number of people who got the service (#23 in Table S1). There were also reports that they have encouraged and even forced the health professionals to provide service for the communities (#24 in Table S1).

## Discussion

This article contributes to a holistic understanding of the extent and range of conflict effects on the health of communities. The quantitative findings provided an empirical view of the effect of conflict on the health of the communities; the qualitative findings provided deeper insights about the health experience of the communities during the conflict. It also sheds light on the complex health challenges faced by patients and their communities, with the coping strategies implemented to attenuate these challenges.

As exemplified by the high number of both direct and indirect morbidity and mortality in the communities, residents’ health declined during the four-month period of conflict. The reported number of deaths resulted in a rate of 15 per 1000 people higher compared to Ethiopia’s total crude death rate (6.67 per 1000 people per year). As the study shows the death rate for only the six-month period and the crude death rate reflects the number of deaths for an entire year, this discrepancy was high. Burnham et al*.* reported 654,965 war-related deaths in Iraq war from 2003 to 2006, more than doubling the pre-war period from 5.5 deaths per 1000 people to 13.3 deaths per 1000 people [23]. Our findings show the impact was much greater. This disparity may be due to the difference in study periods; we included only the six-month active combat period, whereas they include a four-year fatality rate. It may also be due to differences in the setting of the conflict and the conflicting parties. Among reported cases of morbidity and mortality, we found 96% and 75.2%, respectively, to have been due to indirect causes. We also found that the ratio of indirect to direct cause of mortality to be 6:1. Other finding also reported that indirect mortality is considerably higher than direct mortality accounting for at least 75% of total mortality associated with conflict[16].

These consequences show the broader pattern impact of conflict beyond direct killing and shooting. Conflict-related causalities in this study were considerably lower than those reported by Burnham and his colleague in the Iraq war, where violent deaths accounted for 54.8% of total deaths [23]. This difference may be accounted for by the circumstances of the wars; in Iraq, a higher number of air strikes may have increased the number of causalities. In the current study, most of the self-reported cases of illness (48.2%) and death in the family (40.7%) reported occurred in those aged less than 15 years. According to data from the Violation Documentation Center, a dataset that records violent deaths in Syria war from 2011 to 2016, children aged 15 or younger accounted for 23.3% of these deaths by 2016 [10]. This shows that children are more vulnerable to the adverse consequences of conflict-associated interruptions of health services. Multiple studies conducted in Syria, Pakistan, Colombia, Nepal, Afghanistan, Nigeria, Burundi, and Uganda have shown that conflict significantly hampers the utilization of maternal and child health (MCH) service [19, 24–30].

Indirectly, the conflict also caused tremendous consequences for the health of the communities studied, including through a lack of electricity and clean water. These deprivations resulted in outbreaks of communicable diseases such as typhoid and malaria. Similarly, the conflict inflicted famine and food insecurity, which increased the number of adults and children affected by malnutrition. Communities suffered from the collapse of the health system: closure and looting of facilities, displacement of health workers, and disruption of the supply chain. The finding that up to 78.6% of patients did not get any health services during their illness is consistent with studies in Uganda, Liberia, and Syria that reported negative associations between conflicts and the utilization of health services [8, 31–33].

The northern Ethiopian conflict caused many private clinics and pharmacies to close, along with public health services. This contradicts an experience in Somalia where private health services filled a vacuum generated by the absence of public services [34]. The fragmentation of private health actors resulted in further deterioration of the public health system. The results of our quantitative data show that 37.1% of patients have used traditional medicine and home remedies. The quantitative interview reports of health workers providing health services at the communities level using available materials as a measure to cope with the closure of health facilities and shortage of supplies are in line with reports of the Syrian crisis 2011–2017. Health workers there adapted their practices to cope with the shortage of medical supplies [35]. Communities cooperation and support facilitated by religious leaders and volunteer peace committees played stabilizing roles and limited adverse health consequences. This shares some similarities with studies in parts of Africa where the church and its leaders played active roles in stabilizing life for residents in conflict settings [36].

## Study limitations

There were several limitations. We collected data six months after the conflict in the area. As a result, there may be a recall bias. Our data only captures perspectives of people who stayed in the area, leaving out the health experiences of displaced peoples, for whom the conflict may have had just as many, if not more, adverse health consequences. The study only report cases of illness and death over the six-month period without comparison to the pre-conflict period. Had we been able to compare these periods we would have provide a more comprehensive understanding at the effect of conflict on health of the communities. Our study did not assess the psychological and mental health conditions due to difficulties in diagnosing these conditions using retrospective reports from participants. Future studies should also assess the health impacts of conflict on survivors and displaced individuals as these topics were beyond the scope of the present study. Therefore, caution must be exercise when interpreting these findings.

## Conclusions

The northern Ethiopian conflict caused numerous adverse consequences for the health of the communities we studied. The causes were both the direct war-related casualties and the breakdown in the health system and health-supporting structure. The conflict restricted access to health services and resulted in disruption of the health system and infrastructures, which may have a long-term negative health impact. The national health officials and other stakeholders should develop strategies to address the short-term and long-term impacts of the conflict. These strategies should include restoring destroyed facilities, providing a rehabilitation program for survivors of violence, and establishing vaccination programs for children born during the conflict. The coping strategies used by participants in this study offer valuable lessons elsewhere for bolstering communities resilience. Future studies are necessary to strengthen the conclusions.

### Supplementary Information

Below is the link to the electronic supplementary material.

Supplementary file1 (DOCX 43 kb)

## Data Availability

We are not able to share our data publicly due to ethical considerations, but are available from the author by a request to the Woldia University Research and Community service Office/Woldia, Ethiopia (kingyimer@yahoo.com).
