# Supplementary material for: Assessing the health consequences of northern Ethiopian armed conflict, 2022
Source: J Public Health Policy. 2024 Feb 3;45(1):43–57. doi: 10.1057/s41271-023-00464-z (PMC10920422; doi:10.1057/s41271-023-00464-z)
Supplement: Supplementary file 1 — Supplementary file1 (DOCX 43 kb) [file 41271_2023_464_MOESM1_ESM.docx]

**Assessing the health consequences of northern Ethiopia armed conflict, 2022**

Mulugeta Wodaje Arage^1*^, Henok Kumsa^1^, Mulu Shiferaw Alemu^2^, Abebe Tarekegn Kassaw^3^, Efrem Mebratu^4^ Abayneh Tunta^2^, Woldeteklehymanot Kassahun^5^, Amanuel Adissu^6^ , Molla Yigzaw^7^, Tilahun Hailu^8^, Lebeza Alemu Tenaw^8^

^1^School of Midwifery, College of Health Sciences, Woldia University, North Wollo, Amhara Region, Ethiopia

^2^School of Medicine, College of Health Sciences, Woldia University, North Wollo, Amhara Region, Ethiopia

^3^Department of Pharmacy, College of Health Sciences, Woldia University, North Wollo, Amhara Region, Ethiopia

^4^Department of Pharmacy, College of Medicine and Health Sciences, Debre Markos University, Debre Markos, Amhara Region, Ethiopia

^5^Department of Medical Laboratory, College of Health Sciences, Woldia University, North Wollo, Amhara Region, Ethiopia

^6^Department of Public Health, College of Health Sciences, Injibara University, Injibara, Amhara Region, Ethiopia

^7^Department of Public Health, College of Health Sciences, Debre Markos University, Debre Markos, Amhara Region, Ethiopia

^8^School of Public Health, College of Health Sciences, Woldia University, North Wollo, Amhara Region, Ethiopia

^*^Corresponding author: Woldia University, North Wollo, Amhara Region, Ethiopia

Email: [mulewodaje@gmail.com](mailto:mulewodaje@gmail.com)

**Supplementary Material**

**Table S1. Direct quotes from qualitative study participants**

| **Citation #** | **Quotes** | **References** |
| --- | --- | --- |
|  | …each day, civilians had lost their lives due to gunshots. Heavy artillery has attacked their homes. In our kebele alone, there were a total of 63 disease related and 45 conflict related deaths | Health extension worker from Wadla |
|  | *Many people were affected by waterborne diseases such as typhoid, giardiasis, amoebiasis, and other acute gastrointestinal infections. Additionally, many children presented with an eye disease exhibiting symptoms similar to conjunctivitis. I believe this could be linked to air pollution caused by artillery gas.* | Medical doctor, Woldia |
|  | How can I express it? A horrible time that should be condemned in all possible terms. There was almost no problem that hasn’t happened. Every person was in a difficult situation. There was no water to drink and no electricity to cook. Elders were suffering due to a lack of caring and supportive person, children were suffering due to a lack of food; and chronic patients were suffering due to a lack of medication. Young and older women were gang raped everywhere. | Member of peace committee, Guba Lafto |
|  | It was not only the war we were facing; the famine was the scariest; many were losing their lives due to a food shortage. | Pregnant women, Wadla |
|  | *I recall one elder woman for whom I was called by neighbors to give care. When I arrived, I found that she has developed a bedsore in her back and was in critical condition. Later, I learned that she had been living alone for several days after her family fled the area, until her neighbors discovered her situation* | Nurse, Woldia |
|  | I know a 16-year-old girl who was raped at her home in front of her family. After her rape, her father killed himself. | Community elder, Harbu |
|  | …in one of the days, a girl came to me seeking help. She told me the story of her and her friend. The TPLF forces have taken her and her friend into the houses separately and raped them. After the soldiers went out, they talked to each other about what happened to them. But the first girl said the soldiers left her untouched. Then the second girl also lied; she told her friend they left her untouched too. But to the worst, the girl came to me pregnant. This much was how the raped girls hid themselves. | Medical doctor, Harbu |
|  | What was there that the war didn’t cause for us? The feelings of health workers, the shortage of medications, the robbery, looting, and destruction of health facilities—it was due to the invasion that all this happened. | Community elder, Wadla |
|  | *During the conflict, obtaining health care was difficult for the community. The only hospitals that were offering service were Woldia Hospital and our (Lalibela) hospital. Even though, as we were short on professionals and medications, it is difficult to say we were providing service. We were only assisting those with emergency. Moreover, due to the lack of electricity and the restriction of movement at night, this was only possible from 6:00 a.m. to 6:00 P.M* | Nurse, Lalibela |
|  | The health of the community was in danger due to the vandalism, looting, and damage to the health facilities. There was no medicine for chronic disease patients, especially those with diabetes, HIV/AIDS, and hypertension. Some were obliged to buy medication up to 1500 ETB. Many have lost their lives behind closed doors. Others died on the road, trying to go to Dessie to search for medicine. | Pharmacist, Woldia |
|  | After the government troops left the area, there was no one to gourd the hospital. With concern about potential shortage of medications once the juntas (TPLF fighters) took control of the area, some individuals took the opportunity to loot the medications. When the Juntas arrived, they also took the remaining medication | Pharmacist, Woldia hospital |
|  | *At the time, it was difficult to find health professionals and other health workers as they had fled the area due to security concern. Only a few remained, and some have yet to return* | Public health professional, Lalibela |
|  | *One of the most pressing health issues we faced was the use of un-prescribed and expired medications. Since there were no pharmacies or medical supplies available, patients were taking whatever medications they could find without checking the prescription or expiration date. For instance, if someone fell sick with pneumonia and find a typhoid medication in their home or a neighbor’s home, they would take it.* | Health center manager, Lalibela |
|  | It was very challenging to go to health institutions. You could only move during the day from 6 a.m. to 6 p.m. Besides, you can’t be in a group if you have to go; you should only go alone. However, when you go alone, if they get you on the road, you may be robbed and beaten. There are also some women who have been raped when they were going to health institutions. | Nurse, Woldia |
|  | *When the fighting become intense, they bring their injured fighters to the hospital and use it as a shelter from the drone strikes. They also threatened to take over the hospital and turn it into military camp if we did not treat them* | Medical director, Woldia |
|  | *During that time, you had better pray not to get sick because if you did, there was nothing you could do. Your only option was to use traditional medicines and home remedies. Even doing dua (praying) together was not possible as gatherings of more than three people were prohibited* | Religious leader from Guba lafto |
|  | *When our medication was completely finished and many patients were dying due to lack of medications, the religious leaders and peace committees communicate with peoples in Bahr Dar and brought medications using animal power. This saved the lives of many individuals, especially those of chronic patients, who were in urgent need of medications* | Medical doctor from Lalibela |
|  | I myself have tried to provide food by collecting from the community and distributing 3 kilos of flour to the neediest, advising them to use it as MUQ or ATMIT (a local soft food made by boiling flour of wheat or legumes in water until thick) to save food. | Members of the peace committee, Harbu |
|  | The hospital was not functional sometimes and started to be functional after our father organized and encouraged the health professionals to provide service. Later, when the number of mothers who needed surgical operations increased and many died due to a lack of cesarean section service, our father found one surgeon who lived nearby, and by communicating with him and paying him 40 thousand Birr per month from the church money, the hospital started to provide CS services and other surgical operations. | Medical doctor, Lalibela |
|  | *Since most of the health facilities were closed, we were providing home-to-home services in our respective areas. In our kebele, one gynecologist and I were the only available health professionals, and we did our best to serve as much as we could. Even during the night, when there was emergency case such as labouring mothers, the families will come and take us. …by doing like this, we were able to save many lives. Personally, I have assisted around nine labouring mothers* | Health extension worker, Harbu |
|  | *When we were providing home-to-home service, one of the main challenges we were facing was lack of materials and supplies. Mainly, during delivering of labouring mothers, we faced a lack of surgical gloves, and suturing stiches. Our only option at that point was using plastics as surgical gloves and cloth stich’s as surgical stiches* | Midwife, Kobo |
|  | *When the outbreak of diarrhea and typhoid began, each professional took the initiative to educate the community in their respective neighborhood about how to clean the water and keep personal hygiene* | Nurse, Woldia |
|  | After controlling the health facilities, they (TPLF) were providing service using their own health professionals. I know one laboring mother who was about to die due to prolonged labour has delivered safely through an operation (CS) done at the health center by their doctor. But due to a lack of trust, not many patients were going to them. | Female interviewee, Guba Lafto |
|  | They (TPLF fighters) were encouraging and even forcing us to open the health center and give service; however, that was for their own purpose. Firstly, it will help them to get the trust of the community, as they will not be blamed for robing the health facilities and preventing the community from getting service. Secondly, in order to not robe directly by themselves, they will tell us to open the health institution to provide service, but when we open, they will come and take whatever is needed. So, when they get the health center closed, they will be very angry and threaten us. If we don’t provide service, they will take everything and change the health center into their treatment center. So, we have tried to provide service as much as we could with the available professionals. Even if there was nothing we could do, we would not close it. We fear that once we close it, they will take control of it, destroy it, and make it unable to function again. | Midwife, Lalibela |

Others*; Going to holy water places, going to traditional healers

Figure S1. Solutions taken by patients during the armed conflicts in northern Ethiopia, 2022 (n = 224).
